# Potential Role of Hedgehog Pathway in Liver Response to Radiation

**DOI:** 10.1371/journal.pone.0074141

**Published:** 2013-09-16

**Authors:** Sihyung Wang, Youngjae Lee, Jieun Kim, Jeongeun Hyun, Keumju Lee, Younghwa Kim, Youngmi Jung

**Affiliations:** 1 Department of Biological Sciences, Pusan National University, Pusan, Korea; 2 Department of Emergency Medical Technology, Kyungil University, Gyeongsan, Korea; National Cancer Institute, United States of America

## Abstract

Radiation-induced fibrosis constitutes a major problem that is commonly observed in the patients undergoing radiotherapy; therefore, understanding its pathophysiological mechanism is important. The Hedgehog (Hh) pathway induces the proliferation of progenitors and myofibroblastic hepatic stellate cells (MF-HSCs) and promotes the epithelial-to-mesenchymal transition (EMT), thereby regulating the repair response in the damaged liver. We examined the response of normal liver to radiation injury. Male mice were sacrificed at 6 weeks and 10 weeks after exposure to a single dose of 6 Gy and the livers were collected for biochemical analysis. Irradiated (IR) and control mice were compared for progenitors, fibrosis, Hh pathway, and EMT at 6 and 10 weeks post irradiation. Fatty hepatocytes were observed and the expressions of Hh ligand, Indian Hh. were greater in the livers at 6 weeks, whereas expression of another Hh ligand, Sonic Hh, increased at 10 weeks post irradiation. Both Smoothened, Hh receptor, and Gli2, Hh-target gene, were up-regulated at 6 and 10 weeks after irradiation. Accumulation of progenitors (CD44, Pan-cytokeratin, and Sox9) was significant in IR livers at 6 and 10 weeks. RNA analysis showed enhanced expression of the EMT–stimulating factor, tgf-β, in the IR livers at 6 weeks and the upregulation of mesenchymal markers (α-SMA, collagen, N-cadherin, and s100a4), but down-regulation of EMT inhibitors, in IR mouse livers at 6 and 10 weeks. Increased fibrosis was observed in IR mouse livers at 10 weeks. Treatment of mice with Hh inhibitor, GDC-0449, suppressed Hh activity and block the proliferation of hepatic progenitor and expression of EMT-stimulating genes in irradiated mice. Therefore, those results demonstrated that the Hh pathway increased in response to liver injury by radiation and promoted a compensatory proliferation of MF-HSCs and progenitors, thereby regulating liver remodeling.

## Introduction

Radiotherapy has been used for more than 100 years and has become a necessary treatment for a broad range of cancers [1]. Today, it is employed alone or combined with other therapies, such as chemotherapy or surgery, and it improves the cancer cell killing effects of advanced technologies. However, it also damages normal cells, inducing either acute or long-term side effects [1]. Both types of side effects require healing of wounds in the irradiated areas.

The early effects of radiotherapy include DNA damage, which leads to apoptosis and acute inflammatory responses in the irradiated areas. If these effects are not stabilized by the proper treatments, they could be prolonged because of overproduction of inflammatory factors, cytokines, other deleterious factors, such as nitric oxide [2]. Radiation-induced fibrosis is a chronic progressive change seen as a long-term effect of radiotherapy. Radiation promotes the formation of reactive oxygen species (ROS) [3], [4] which induce the dysregulated activation of myofibroblastic hepatic stellate cells (MF-HSCs) by increasing the level of TGF (transforming growth factor)-β1 [5], TGF-β1 is a well known cytokine that induces the profibrotic pathway and fibrosis in damaged organs including liver [6]. Hence, investigation of radiation-induced damage is very important because it can explain the pathophysiological features of early and late effects of radiotherapeutic injuries. The aim of the present study was to investigate the effects of radiation on healthy liver tissues.

The hedgehog (Hh) pathway is an essential morphogene for embryogenesis and tissue remodeling in adult tissue. Hh ligands, Shh (Sonic Hh), Ihh (Indian Hh), and Dhh (Desert Hh), bind to the Hh receptor, Ptc (patch), which releases Smo (smoothened; other type of receptor) into the cytosol. Released Smo promotes the translocation of cytoplasmic Glis (glioblastoma family: Gli1, Gli2, Gli3) into the nucleus, and nuclear Glis acts as a transcriptional factor, activating Hh signaling [7], [8], [9]. Emerging evidence shows that Hh signaling is activated in damaged liver, where it regulates tissue reconstruction. The level of Hh expression was shown to parallel the stages of liver disease [10], especially the degree of fibrosis. Recent studies demonstrated that apoptotic hepatocytes in patients and experimental animals with chronically damaged livers produced Hh ligands, which promoted the expansion of progenitors and induced the EMT (epithelial-to-mesenchymal transition) [11], [12]. In addition, Hh signaling is known to activate the transformation of quiescent hepatic stellate cells (Q-HSC) into myofibroblasts (MF)-HSCs [13]. Thus, Hh signaling is critically important in hepatic fibrogenesis [10], [13], [14], [15], [16], [17].

Given that irradiation leads to apoptosis and fibrosis in patient livers and that Hh produced in the injured livers is a key factor regulating fibrosis, we hypothesized that Hh signaling might be related to defective wound healing that induces the fibrosis seen in irradiated tissues or organs. To prove our hypothesis, we evaluated whether Hh signaling was activated during liver damage caused by low dose irradiation and whether this activated Hh signaling contributed to compensatory hyperplasia of hepatic progenitors and/or myofibroblasts, thereby leading to hepatic fibrogenesis. Our results demonstrated that activation of the Hh pathway occurs at 6 weeks post irradiation and persists until 10 weeks after irradiation. Increased expression of Hh signaling promotes proliferation of progenitors and activation of HSCs into MF-HSCs, eventually contributing to hepatic fibrogenesis.

## Materials and Methods

### Animal Studies

Male C57BL6 mice at 6 weeks old were purchased from Hyochang (Dae-gu, Korea), fed with normal diet, watered, and housed with a 12 h light-dark cycle. In vivo experiments were performed as previously described [18]. Mice were 7 weeks of age and weighed an average of 21 g at the start of the experiments. To induce liver injury, the mice were subjected to a single irradiation with 6Gy onto whole body. Head and lower part of abdomen of mice were protected by the customized lead shield. Mice were sacrificed at 6 weeks and 10 weeks post irradiation. Before irradiation, mice were anesthetized by zoletil 50 (5 mg/kg body weight, Virbac S.A, France) to immobilize in the recumbent position on a treatment table. Liver tissue was collected for histological and biochemical analysis.

Animal care and surgical procedures were approved by the Pusan National University Institutional Animal Care and Use Committee and carried out in accordance with the provisions of the NIH Guide for the Care and Use of Laboratory Animals.

### Hh Inhibitor, GDC-0449, Treatment

To investigate the effect of GDC-0449 (Selleck Chemicals, Houston, TX), in the healthy liver, 8 mice were treated with DMSO (n = 4) or 25 mg/kg GDC-0449 (n = 4).The dose of GDC-0449 was decided based on the previous study [19]. GDC-0449 was freshly constituted daily in DMSO. In the injury model, GDC-0449 (n = 5) or DMSO (n = 4) was injected into mice by intraperitioneal injection (IP) 2 hours before getting radiation. After radiation treatment, mice were given a daily of 25 mg/kg GDC-0449 or DMSO for 6 weeks and sacrificed to collect tissue and serum. No death was observed in those animal experiments.

### Liver Histology and Immunohistochemistry

Liver specimens were fixed in 10% neutral buffered formalin, embedded in paraffin and cut into 4 µm sections. Specimens were dewaxed, hydrated, and stained usual method with standard hematoxylin and eosin (H&E) to examine morphology and Sirius Red to assess fibrosis. For immunohistochemistry (IHC), sections were incubated for 10 min in 3% hydrogen peroxide to block endogenous peroxidase. Antigen retrieval was performed by heating in 10 mM sodium citrate buffer (pH 6.0) for 10 min or incubation with pepsin for 10 min. Sections were treated with Dako protein block (X9090; Dako Envision, Dako) for 30 min and incubated with primary antibodies, Ihh (ab39634; Abcam), Pan-CK (Z0622; Dako), CD44 (550538; BD Biosciences), Sox9 (AB5535; Millipore), or α-Sma (ab5694; Abcam) at 4°C overnight. Other sections were also incubated at 4°C overnight in non-immune sera to demonstrate staining specificity. Polymer horseradish peroxidase (HRP) anri-rabbit (K 4003; Dako) or anti-rat IgG-HRP (sc-2006; Santa Cruz Biotechnology) was used as secondary antinody. 3,3′-Diaminobenzidine (DAB) was employed in the detection procedure.

### Cell Counting

To quantify the number of Ihh, CD44, Pan-CK, and Sox9-positive cells, 10 portal tract (PT) areas were randomly selected per section at ×40 magnification for each mouse. After excluding the major bile duct in each portal tract (PT) from consideration, cells staining positively for Ihh, CD44, Pan-CK, Sox9 in 10 PTs per slide were counted at ×40 magnification. PT chosen for analysis contained portal vein that ranged from 110 to 170 µm. The Ihh, CD44, Pan-CK, Sox9-positive cells were quantified by counting the total number of Ihh or CD44 or Pan-CK or Sox9-positive cells per field and dividing by the total number of hepatocytes per field.

### Quantitative Real-time PCR

Total RNA which had been stored at −80°C was extracted with TRIZOL™ (Ambion® by Life technologies). After assuring RNA quality and concentration, gene expression was evaluated by QRT-PCR analysis. mRNAs were quantified by real-time RT-PCR per the manufacturer’s specifications (Eppendorf, Mastercycler Real-Time PCR). The sequences of primers for mice are listed in Table 1. Samples were analyzed in duplicate according to the ΔΔCt method. All PCR products were directly sequenced for genetic confirmation in Macrogen Inc (Korea).

**Table 1:** Sequences of primers used for QRT-PCR.

Gene	Forward Sequence	Reverse Seuquence
9 s	GACTCCGGAACAAACGTGAGG	CTTCATCTTGCCCTCGTCCA
Ihh	GAGCTTTCCAGGTCATCGAG	TGATTGTCCGCAATGAAGAG
Shh	GGAACTCACCCCCAATTACA	TGCACCTCTGAGTCATCAGC
Smo	CAGCAAGATCAACGAGACCA	AAGTGGCAGCTGAAGGTGAT
Gli2	CAAGCAGAACAGCGAGTCAG	CCTCAGCCTCAGTCTTGACC
TGF-β1 -	TTGCCCTCTACAACCAACACAA	GGCTTGCGACCCACGTAGTA
α-SMA	AAACAGGAATACGACGAAG	CAGGAATGATTTCCAAAGGA
Collagen *α1*	GAGCGGAGAGTACTGGATCG	GCTTCTTTTCCTTGGGGTTC
N-cadherin	CAGTGGACATCAATGGCAATCA	CATTTGGATCATCCGCATCA
s100a4	AAAGAGGGTGACAAGTTCAA	CGGGGTTCCTTATCTGG
Bmp7	GTGGTCAACCCTCGGCACA	GGCGTCTTGGAGCGATTCTG

### Western Blot Assay

Total protein was extracted from freeze-clamped liver tissue sample that had been stored at −80°C. Whole tissues were homogenized in RIPA (78510; Thermo) supplemented with protease inhibitors (Complete Mini 11 836 153 001; Roche). Equal amount of total protein (150 ug) were fractionated by polyacrylamide gel electrophoresis and transferred to PVDF (polyvinylidene difluoride) membranes. Primary antibodies against Shh (sc-9024; Santa Cruz Biotechnology), Ihh (ab39634; Abcam), Smo (ab72130; Abcam), Gli2 (GEN-18-272-197571; Genway), TGF-β (3711S; Cell signaling), and α-Sma (A5228-200UL; Sigma-aldrich) were used in this experiment. Membranes were developed by chemiluminescence (ATTO Corporation). The blots that were obtained from three independent experiments were scanned and an ROI around the band of interest was defined. Band intensities were calculated by using CS analyzer 2.0 program (ATTO Corporation).

### Triglyceride Measurement

Total liver triglycerides were measured by using Triglyceride Fluorometric Assay Kit from Cayman Chemical (Nashville, Tennessee) following the manufacturer’s specifications.

### Measurement of AST/ALT

Serum aspartate aminotransferase (AST) and alanin aminotransferase (ALT) were measured by using ChemiLab GOT/GPT (IVD Lab Co., Korea) according to the manufacturer’s instructions.

### Statistical Analysis

Results are expressed as the mean±SD. Statistical differences were determined by Student’s t-test. P-values <0.05 were considered to be statistically significant.

## Results

### Increased Expression of the Hedgehog Pathway in the Livers of Irradiated Mice

**Figure 1:**
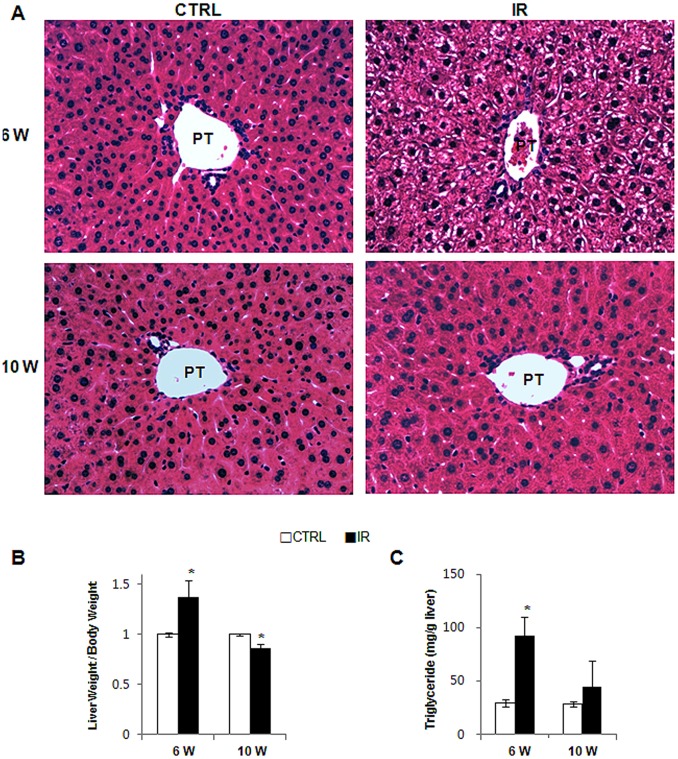
Changes of liver morphology and weight of the irradiated mice. (A) H & E staining shows fat accumulation in hepatocytes in liver from representative radiation-treated mice. (B) Relative liver weight/body weight of mice (*p<0.05 vs own control group) (PT: portal tract, CTRL : control group, IR : irradiation group).

Liver sections from radiation-treated mice were examined for liver injury using H&E staining. Fat accumulation in hepatocytes of the portal tract was observed in the liver from IR mice, but was not seen in livers from control mice at 6 weeks post irradiation (Figure 1A, top panel). Striking increase in the liver weight/body weight (W/BW) ratio (1.37±0.167, compared to CTRL) confirmed the fat accumulation in IR mice with fatty hepatocytes (Figure 1B and Table S1). In addition, triglyceride assay supported the greater increase of fat in irradiated liver at 6 weeks (Figure 1C). However, the LW/BW ratio declined in the IR group compared to control group at 10 weeks after irradiation (0.86±0.045, compared to CTRL). In addition, fatty hepatocytes were rarely detected in the liver at 10 weeks post radiation and these livers appeared grossly normal at baseline (Figure 1A, bottom panel).

**Figure 2:**
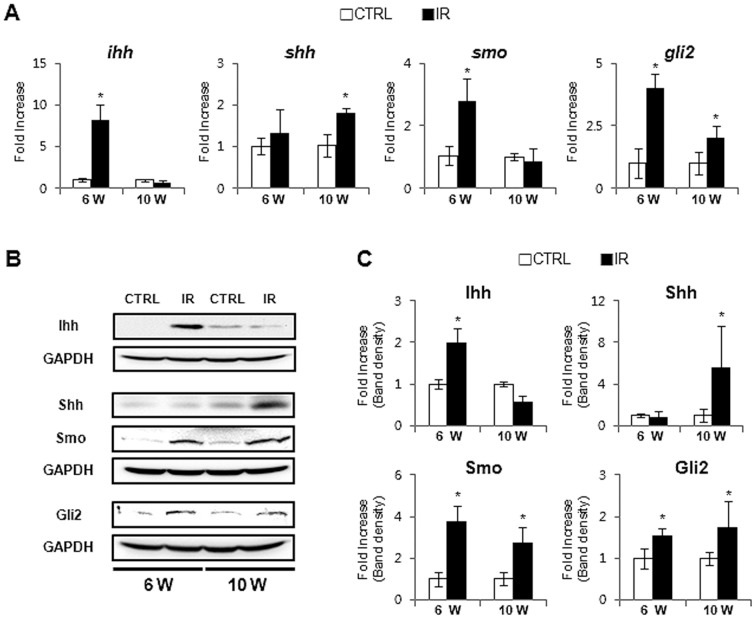
Enhanced activation of the Hh signaling pathway in the liver post irradiation. (A) QRT-PCR analysis of liver mRNA from radiation-treated mice for ihh, shh, smo, and gli2 ((n ≥3 mice/group) Mean±SD results are graphed. (B) and (C). Western blot analysis of Ihh, Shh, Smo, and Gli2 (GAPDH was used as an internal control). Data shown represent one of three experiments with similar results (B: Immuoblot/C: Band density) (n ≥3 mice/group). Data represent the mean ± SD of three independent experiments (*p<0.05 vs own control group).

**Figure 3:**
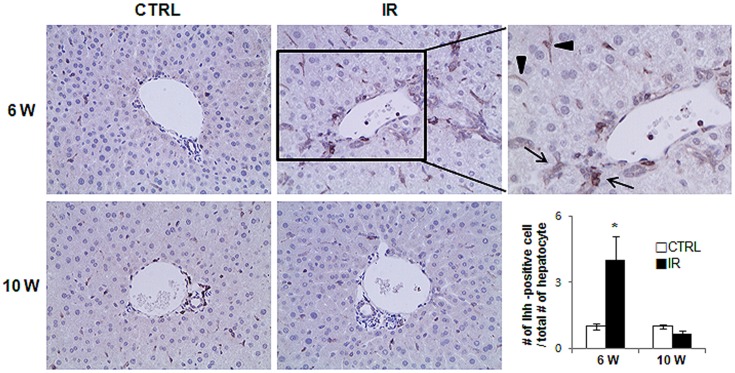
Ihh expression in the injured livers of mice by radiation. IHC for Ihh in the liver at 6 and 10 weeks post radiation. Brown color indicated Ihh-positive cells. Magnified representative image from IR livers at 6 weeks (x63) is shown in right panel (arrow and arrowhead indicate the oval cell and HSC-looking cells, respectively). The number of Ihh-postive cells were counted and graphed (*p<0.005 vs own control group).

The LW/BW increased at 10 weeks compared to 6 weeks (Figure 1B) in control group, but the reduction in the LW/BW in the IR mice at 10 weeks suggested that the repair response to radiation injury might shrink the liver volume. Emerging evidence demonstrates that Hh signaling is the critical pathway regulating liver fibrosis [20]. Recent studies showed that the Hh pathway is highly expressed in the chronic liver diseases [13], [14]. Thus, we hypothesized that Hh signaling pathway might be activated in the liver of irradiated mice at 6 weeks and might contribute to the fibrosis in livers of these mice that was evident at 10 weeks. We examined Hh expression at 6 weeks and observed a significant increase in Hh signaling molecules in the livers of irradiated mice. RNA expression of the Hh ligand, ihh, the Hh receptor, smo, and the Hh-target gene, gli2, showed a greater increase in the IR group than in the control group. Ihh expressions were decreased in the livers of IR mice, but another Hh ligand, shh, was up-regulated in IR mice at 10 weeks (Figure 2A). Western blot assays confirmed the increase of Hh signaling molecules in the livers of IR mice at both 6 and 10 weeks (Figure 2B–C). The IR mouse livers showed upregulation of Ihh at 6 weeks and downregulation at 10 weeks, while Shh expression was increased at 10 weeks, in line with the RNA results. Although a switch in expressions of the Hh ligands, Ihh and Shh, was evident, these expressions seemed to maintain the activation of Smo and Gli2 in the livers from IR mice. Immunohistochemical (IHC) staining for Ihh revealed positive staining in hepatic stellate or progenitor-like cells in the IR group (Figure 3). More Ihh-positive cells were found in the IR mouse livers than in control mouse livers at 6 weeks (Figure 3). Thus, Hh signaling was activated in the damaged liver by irradiation and the increased expression of the Hh pathway suggested its involvement in the repair response of the liver.

### Increased Expansion of Progenitors During Liver Damage

**Figure 4:**
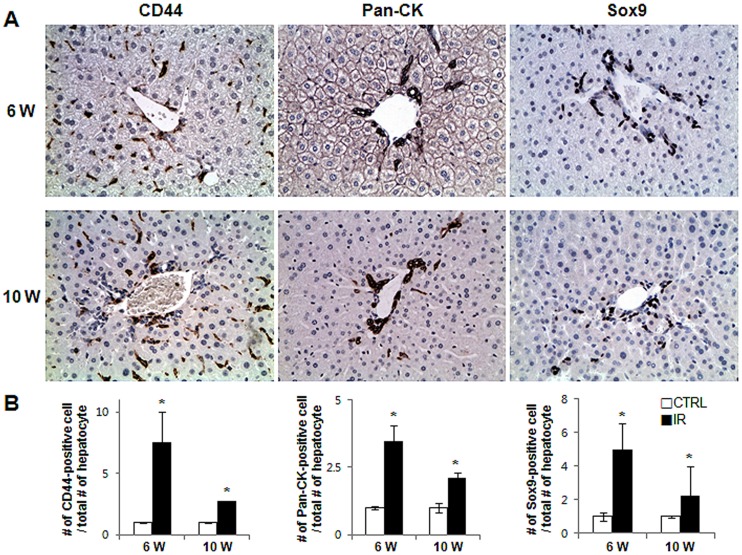
Accumulation of liver progenitors in the radiation-damaged livers. (A) Accumulation of CD44, Pan-Ck, and Sox9-positive progenitor cells in mouse livers from representative radiation-treated mice (X40). (B) The number of CD44, Pan-Ck, and Sox-9-postive cells were counted and graphed (*p<0.05 vs own control group).

The Hh pathway is known to regulate the proliferation of progenitors after liver injury [14]. Therefore, we stained livers from the IR and control groups for CD44, Pancytokelatin (Pan-Ck), and Sox9, three different markers of liver progenitors. Liver injury due to IR induced a greater accumulation of CD44 and Sox9-positive cells than in control mice at both 6 and 10 weeks (CD44∶ 7.55±2.488 and 2.75±0.013, Sox-9∶ 4.995±1.536 and 3.01±1.022 at 6 and 10 weeks, IR compared to control, respectively). Similarly, greater numbers of Pan-Ck-positive cells were seen in IR mice than in control mice (3.46±0.594 at 6 weeks and 2.10±0.195 fold increase at 10 weeks compared to control) (Figure 4 and Figure S1). The number of progenitors seemed to be higher in the IR group at 6 weeks than at 10 weeks, but this difference was not statistically significant. Overall, enhanced expression of Hh appeared to promote a compensatory expansion of the progenitor population after liver injury.

### Increased Fibrosis in Irradiated Mouse Liver

**Figure 5:**
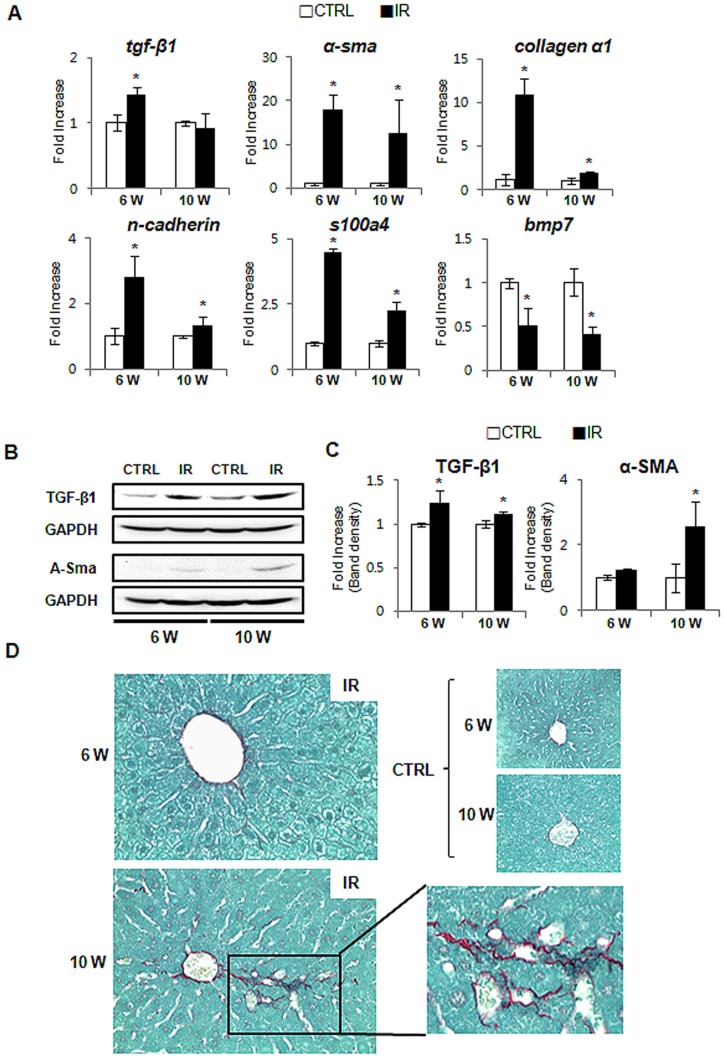
Increased fibrosis and EMT in the irradiated liver. (A) QRT-PCR analysis of liver mRNA from radiation-treated mice for tfg- β, α -sma, collagen a1, n-cadherin, s100a4, and bmp7(n ≥3 mice/group). Mean±SD results are graphed. (B) & (C). Western blot analysis of TGF-β (25 kDa: processed form) (inducer of fibrosis) and α-SMA (fibrogenic marker) expression (GAPDH was used as an internal control) (n≥3 mice/group). Data shown represent one of three experiments with similar results (B: Immunoblot/C: Band density of TGF-β and α-SMA). Data represent the mean ± SD of three independent experiments. (D) Sirius red staining in liver sections from representative control and irradiated mice (X40) (*p<0.05, vs own control group).

The Hh ligand-producing cells release these factors as membranous particles, and this process increases during apoptosis [11], [10]. The HSCs that reside in the space of Disse are in close proximity to hepatocytes, so they are proximal targets of the Hh ligands being produced in injured livers. Quiescent HSCs (Q-HSCs) are activated and transformed into MF-HSCs, which generate collagen fibrils and up-regulate genes related to ECM formation [13]. Several signaling molecules, such as TGF-β and Hh, promote the transformation of Q-HSCs into MF-HSCs through EMT [13]. Accordingly, increased activation of the Hh pathway may induce EMT and promote fibrogenesis in the radiation-treated livers. To assess this possibility, we examined the expression of EMT-related genes and demonstrated that a profibrogenic cytokine (TGF-β) [21], a marker of MF-HSCs (α-smooth muscle actin; α-SMA) [22], and EMT markers (s100a4 [15], N-cadherin [23], collagen α1 [15]) were greatly increased in the livers of the IR group, compared to the control group. In addition, the expression of bmp7, a potent inhibitor of EMT [15], was greatly reduced in the IR group, compared to the control group (Figure 5A). The expression of TGF-β1 at the protein level was upregulated in the damaged livers at both 6 and 10 weeks. The irradiated liver showed higher expression of α-SMA at 10 weeks after damage (1.22±0.05 at 6 weeks and 2.557±0.788 fold increase at 10 weeks) (Figure 5B–C). Sirius red staining demonstrated that more pericellular and sinusoidal deposition of collagen fibrils occurred in IR mice than in control mice at 10 weeks (Figure 5D). Therefore, these results suggested that the activation of the Hh pathway might induce the EMT and promote fibrogenesis in mice whose livers were injured by irradiation.

### Decreased Hepatic Hh Activity in Irradiated Mice with GDC-0449, Smoothened Inhibitor

**Figure 6:**
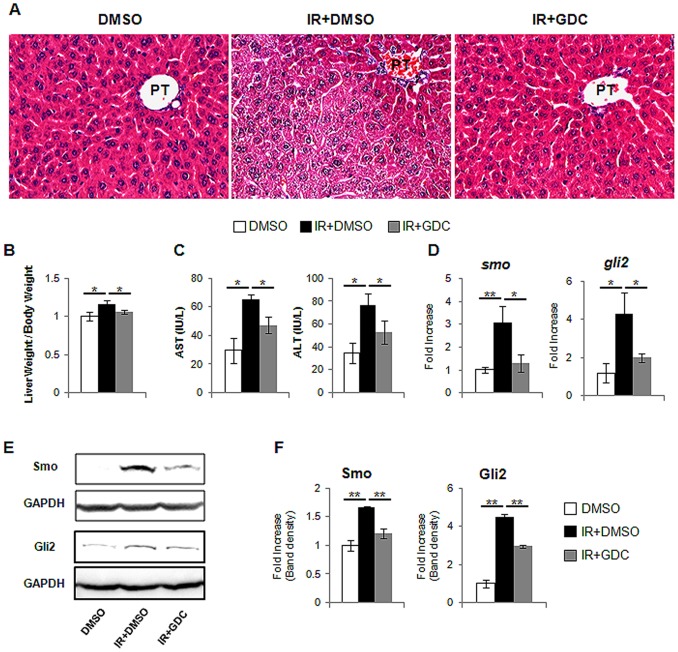
Hh inhibitor, GDC-0449, blocks hepatic Hh activity in the irradiated mice. (A) H&E staining shows less fat accumulation in hepatocytes in liver from representative irradiated mice with GDC-0449 (IR+GDC) (X40). (B) Relative liver weight/body weight of mice. (C) The values of AST and ALT are graphed. (D) QRT-PCR analysis of liver mRNA from DMSO (DMSO), radiation treated mice with (IR+GDC) or without GDC-0449 (IR+DMSO) for smo, and gli2 ((n ≥4 mice/group). Mean±SD results are graphed. (E) and (F). Western blot analysis of Smo, and Gli2 (GAPDH was used as an internal control). Data shown represent one of three experiments with similar results (E: Immuoblot/F: Band density) (n ≥4 mice/group). Data represent the mean ± SD of three independent experiments (*p<0.05, **p<0.005).

We first examined the effect of GDC-0449 or vehicle in the healthy livers. Mice were given a daily intraperitioneal injection (IP) of 25 mg/kg GDC-0449 (N = 4) or DMSO vehicle (n = 4) for 6 weeks and then sacrificed. We did not find any morphological change in the livers from GDC-0449 or vehicle treated mice, compared to non-treated mice (Figure S 2A). In addition, there were no statistical differences in the liver & body weight and Hh activity among these groups (Figure S 2B–D and Table S2). Based on the data for the uninfluential action of GDC-449 and vehicle in the healthy livers, we treated irradiated mice with GDC-0449 (IR+GDC group) or vehicle (IR+DMSO group) for 6 weeks. The livers from IR+GDC group had less fatty hepatocytes (Figure 6A). The increase in the liver weight/body weight (LW/BW) ratio was also ameliorated in GDC-0449 treated mice (IR+GDC), compared to IR+DMSO group (IR+DMSO: 1.16±0.09 and IR+GDC: 1.06±0.03, compared to DMSO) (Figure 6B and Table S2). IR+DMSO-treated mice had elevated serum AST and ALT, whereas IR+GDC-treated mice had alleviated AST and ALT (AST: IR+DMSO- 65.48±3.36vs IR+GDC-47.38±5.99/ALT: IR+DMSO-76.22±10.56 vs IR+GDC-53.07±10.28). Mice which were given a daily of DMSO showed the normal range of AST and ALT (AST: 29.61±8.62/ALT: 34.83±9.01) (Figure 6C). The expression of smo and gli2 gene in IR+GDC group decreased baseline smo and gli2 expression in healthy liver of DMSO-treated mice without irradiation (Figure 6D). The protein expression of both Smo and Gli2 was also reduced in IR+GDC group (Figure 6E–F). Those data demonstrated that GDC-0449 inhibited the activation of Hh signaling in the irradiated livers.

### Suppressed Hh Activity Attenuates the Proliferation of Hepatic Progenitor and Fibrosis

**Figure 7:**
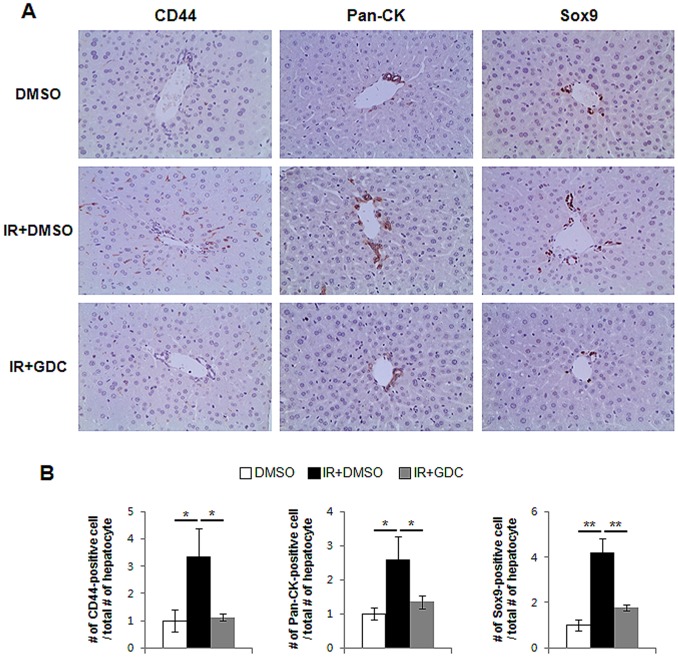
GDC-0449 Inhibits expansion of progenitor in damaged liver by radiation. (A) Decreased accumulation of CD44, Pan-Ck, and Sox9-positive progenitor cells in mouse livers from representative radiation-treated mice which were injected with GDC-0449 for 6 weeks (X40). (B) The number of CD44, Pan-Ck, and Sox-9-postive cells were counted and graphed (*p<0.05, **p<0.005).

**Figure 8:**
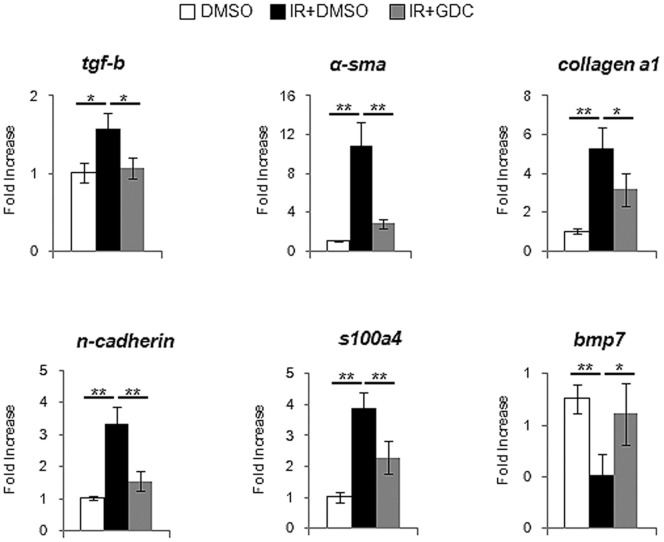
GDC-0449 treatment reduces the expression of EMT-stimulating genes in irradiated livers. QRT-PCR analysis of liver mRNA from radiation-treated mice for tgf-β, α-sma, collagen α1, n-cadherin, s100a4, and bmp7(n ≥4 mice/group). Mean±SD results are graphed (*p<0.05, **p<0.005).

To assess whether the increased Hh signaling directly influenced the proliferation of progenitor and progression of EMT, we treated the irradiated mice with GDC-0449 for 6 weeks. IHC staining for the hepatic progenitor markers clearly demonstrated that the accumulation of progenitors was significantly reduced in the livers from IR+GDC group, compared to IR+DMSO group (Figure 7). The RNA expression of tgf-β and α-sma was greatly up-regulated in the irradiated livers, whereas both genes were down-regulated in the irradiated livers with GDC-0449 treatment, showing the baseline tgf-β and α-sma expression in healthy livers (DMSO group). In addition, the expression of EMT-stimulating factors, s100a4, N-cadherin, collagen α 1, was lower in IR+GDC than IR+DMSO group. EMT-inhibitor, bmp7, was restored to the level of bmp7 expression in DMSO group (Figure 8). Therefore, those results demonstrated that the inhibited Hh signaling abrogated both expansion of progenitors and EMT progression in the irradiated livers.

## Discussion

Radiation therapy is an essential treatment for several malignant tumors as well as for abdominal diseases. However radiotherapy presents practical difficulties in adjusting the exact dosage and timing of irradiation and in ensuring target cell-specific killing. Moreover, the damage to normal tissues after irradiation remains a serious disadvantage of this therapy. Acute effects occur within 10–14 days after starting treatment and can be resolved with appropriate care, but they may be permanent or progressive for many years after the course of radiotherapy.

Long-term effects can emerge after more than 3 months after the end of treatment and include necrosis, atrophy, fibrosis, vascular damage, and carcinogenesis. Both acute and long-term effects of radiotherapy may be progressive and may cause fibrosis because of abnormal responses of irradiated normal tissues. In response to cell death, ischaemia, and inflammation within irradiated tissues, cells regenerate and inflammatory response increases during tissue injury by radiation. Assessing these changes in normal tissues after radiation is very important for understanding the pathophysiological mechanism of fibrosis frequently observed in radiation injury. In the previous experiment, mice were irradiated with 20Gy and showed 70% mortality for one week and 100% for 10days [24]. Fibrosis is the outcome of chronic side-effects of radiation, and we need the living animal for a certain period time, at least 8 weeks, to detect physiopathological changes occurring for a long course. In the present study, we examined the response of normal liver to radiation damage over a time course and provided evidence for the accumulation of fatty hepatocytes and an increase in the EMT process, as well as compensatory proliferation of progenitors at 6 weeks post irradiation. We also demonstrated that these effects of radiation continued until 10 weeks after irradiation, showing the increased fibrosis in livers.

Recent studies demonstrated that increased expression of Hh signaling promotes the proliferation and differentiation of MF-HSCs and progenitors, which contribute to fibrogenesis, thereby orchestrating tissue reconstruction of the damaged liver. Increases in Hh ligands following liver injury (or culture) permit the transition of Q-HSCs into MF-HSCs. The Hh ligands enhance the viability and proliferative activity of MF-HSCs and progenitors, which themselves produce and release Hh ligands, further enriching the hepatic microenvironment with these factors during the accompanying hepatic fibrosis. We demonstrated that Hh signaling regulated the response of liver to radiation, leading to hepatic fibrosis by promoting the EMT. The effect of Hh signaling in the irradiated liver was supported by Hh inhibitor, GDC-0449, treatment; GDC-0449 blocked the hepatic Hh activity and this suppressed Hh signaling leaded to the reduced proliferation of progenitor and fibrosis in the irradiated livers.

In the previous study, 20 or 40 mg/kg of GDC-0449 was tested and both doses successfully inhibited Hh signaling in liver [19]. Even high dose (40 mg/kg) of GDC-0449 treatment did not show the statistical difference from DMSO vehicle treatment and was well tolerated by mice with advanced liver disease. However, these dose-finding tests were conducted in mice at a short course of treatment. In the current study, GDC-0449 injection was needed for a long course, 6 weeks, to investigate the role of Hh signaling in the liver remodeling after radiation treatment. Considering these points, mice were assigned to treatment with 25 mg/kg of GDC-0449 via daily IP injection for 6 weeks.

Increased expressions of the Hh receptor, Smo, and the Hh-target gene, Gli2, were observed in the livers of radiation-treated mice. The Hh ligands, Ihh and Shh, showed differential expression: Ihh expression was higher at 6 weeks, whereas Shh activation was higher at 10 weeks. This difference in the time of expression of Ihh and Shh indicated that the Hh activators, Smo and Gli2, might be transiently activated. This sequential expression of Hh ligands also indicated that Ihh and Shh might differ in their effects on the response of the liver to radiation. Since Shh is known to promote the activation of MF-HSCs [25], the upregulated expression of Shh in IR mice seems to promote proliferation of myofibroblasts, thereby accelerating fibrosis with the action of Ihh.

Although hepatocytes in the healthy livers are quiescent, they enter the cell cycle and repopulate the damaged tissue. However, when massive apoptosis of hepatocytes occurs due to severe or chronic injury, the replicative ability of these cells is blocked and leads to compensatory proliferation of HSCs or progenitors. In the present study, proliferation of progenitors increased in the livers of IR mice, in parallel with the increased expression of Hh signaling. Interestingly, remarkable increase in progenitor proliferation appeared in the IR mice at 6 weeks, although the progenitor accumulation in the livers did not differ significantly between 6 and 10 weeks after irradiation. Earlier activation of Ihh therefore might have influenced the proliferation of progenitors in the IR group. In addition, Ihh up-regulation seems to initiate the EMT process because the relative expression of EMT-related genes was greater in the liver at 6 weeks post irradiation than in the controls. Increased protein expression of SMA and increased collagen deposition, as determined by Sirius red staining, indicated that Shh might participate in activation of HSCs into MF-HSC in the livers at 10 weeks. However, further study is needed to determine the specific actions of Shh and Ihh in individual cell types in the irradiated livers.

In conclusion, those results demonstrated that the expression of the Hh pathway increased in the liver in response to radiation injury and promoted the compensatory proliferation of MF-HSCs and progenitors, thereby influencing liver remodeling. Our findings suggest a novel mechanism for the repair response occurring in response to radiotherapeutic injury, and this new understanding may help to improve the therapeutic effects of radiotherapy.

## Supporting Information

Figure S1 **IHC staining for CD44, Pan-CK, and Sox9 in liver from representative control mice (X40).**
(DOCX)

Figure S2 **Smo inhibitor, GDC-0449, rarely influences the healthy liver.** (A) H&E-stained liver section from representative control (CTRL), DMSO-(DMSO) and GDC-0449- treated mice. (X40) (B) Relative liver weight/body weight of mice. (C) QRT-PCR analysis of liver mRNA from CTRL, DMSO, GDC-0449-treated mice for smo, and gli2 ((n ≥3 mice/group) Mean±SD results are graphed. (D) and (E). Western blot analysis of Smo, and Gli2 (GAPDH was used as an internal control). Data shown represent one of three experiments with similar results (D: Immuoblot/E: Band density) (n ≥3 mice/group). Data represent the mean ± SD of three independent experiments.
(DOCX)

Table S1 **Liver and body weight.**
(DOCX)

Table S2 **Liver and body weight in GDC-0449 treatment.**
(DOCX)
