# Supplementary material for: Potential Role of Hedgehog Pathway in Liver Response to Radiation
Source: PLoS One. 2013 Sep 16;8(9):e74141. doi: 10.1371/journal.pone.0074141 (PMC3774612; doi:10.1371/journal.pone.0074141)

**Supporting Figure S1**. **IHC staining for CD44, Pan-CK, and Sox9 in liver from representative control mice (X40).**


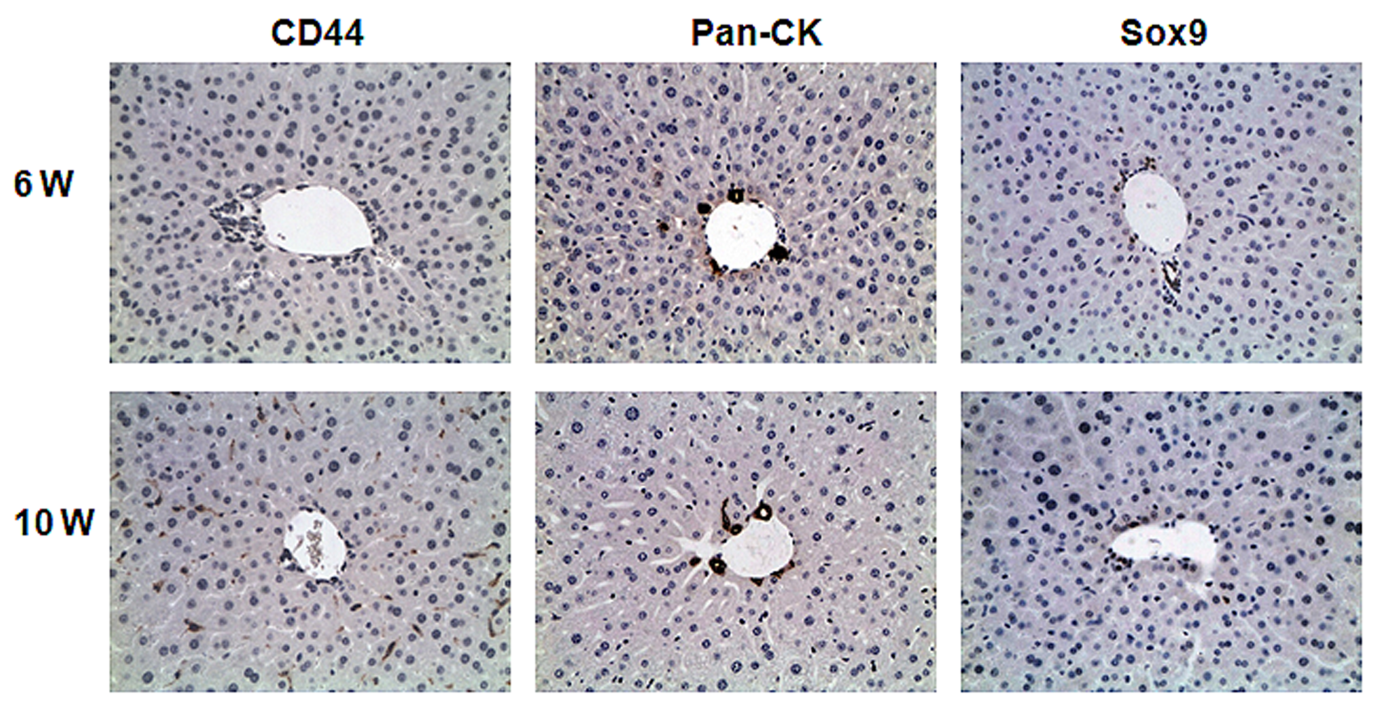

Supplement: Figure S1 — IHC staining for CD44, Pan-CK, and Sox9 in liver from representative control mice (X40). (DOCX) [file pone.0074141.s001.docx]
