# Supplementary material for: Potential Role of Hedgehog Pathway in Liver Response to Radiation
Source: PLoS One. 2013 Sep 16;8(9):e74141. doi: 10.1371/journal.pone.0074141 (PMC3774612; doi:10.1371/journal.pone.0074141)
Supplement: Figure S2 — Smo inhibitor, GDC-0449, rarely influences the healthy liver. (A) H&E-stained liver section from representative control (CTRL), DMSO-(DMSO) and GDC-0449- treated mice. (X40) (B) Relative liver weight/body weight of mice. (C) QRT-PCR analysis of liver mRNA from CTRL, DMSO, GDC-0449-treated mice for smo, and gli2 ((n ≥3 mice/group) Mean±SD results are graphed. (D) and (E). Western blot analysis of Smo, and Gli2 (GAPDH was used as an internal control). Data shown represent one of three experiments with similar results (D: Immuoblot/E: Band density) (n ≥3 mice/group). Data represent the mean ± SD of three independent experiments. (DOCX) [file pone.0074141.s002.docx]

**Supporting Figure S2. Smo inhibitor, GDC-0449, rarely influences the healthy liver.**


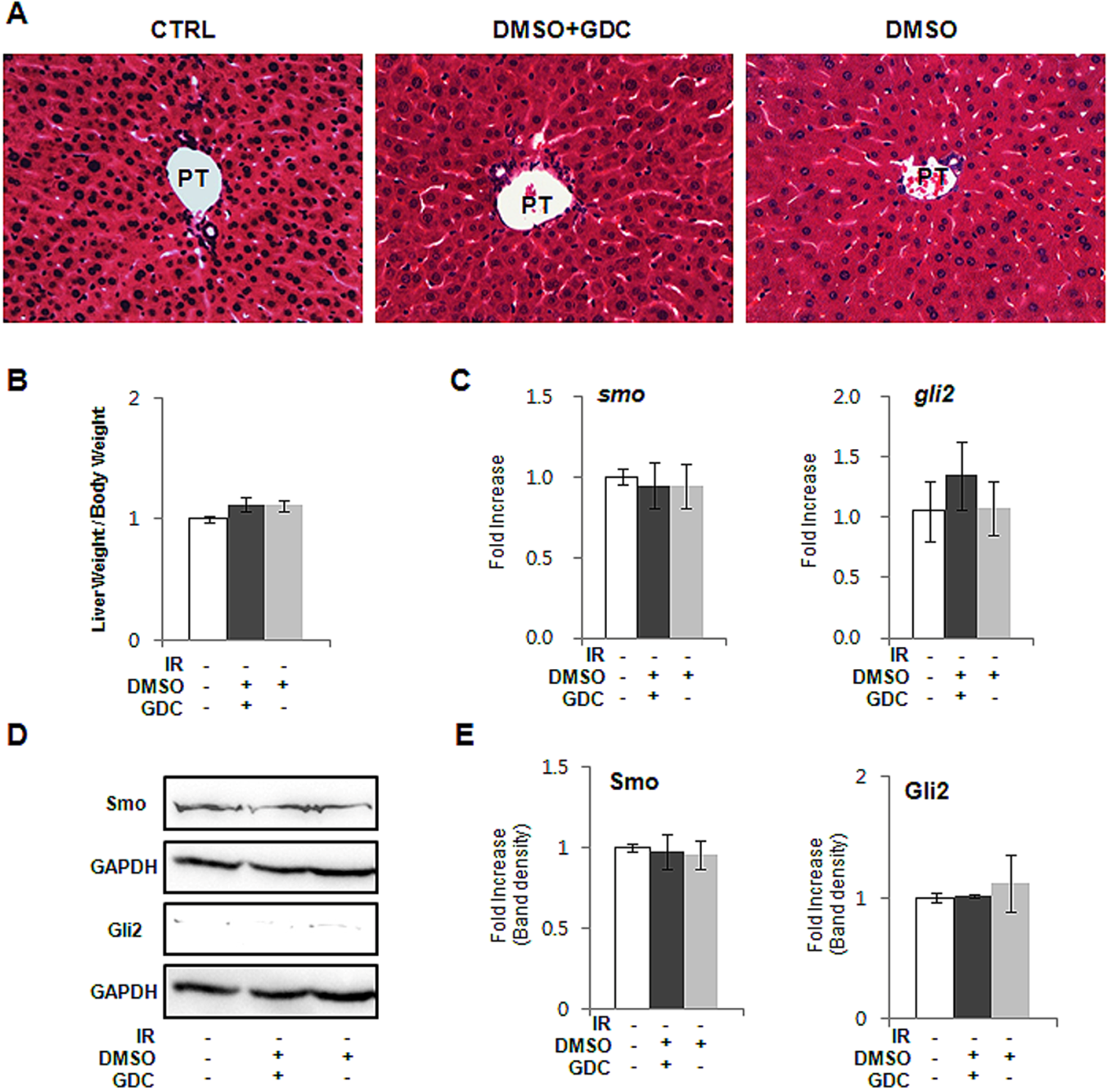


(A) H&E-stained liver section from representative control (CTRL), DMSO-(DMSO) and GDC-0449- treated mice. (X40) (B) Relative liver weight / body weight of mice. (C) QRT-PCR analysis of liver mRNA from CTRL, DMSO, GDC-0449-treated mice for smo, and gli2 ((n ≥ 3 mice / group) Mean±SD results are graphed. (D) and (E). Western blot analysis of Smo, and Gli2 (GAPDH was used as an internal control). Data shown represent one of three experiments with similar results (D: Immuoblot/ E: Band density) (n ≥ 3 mice / group). Data represent the mean ± SD of three independent experiments.
