# Supplementary material for: Potential Role of Hedgehog Pathway in Liver Response to Radiation
Source: PLoS One. 2013 Sep 16;8(9):e74141. doi: 10.1371/journal.pone.0074141 (PMC3774612; doi:10.1371/journal.pone.0074141)
Supplement: Table S1 — Liver and body weight. (DOCX) [file pone.0074141.s003.docx]

**Supporting table S1. Liver and body weight**

|  | | **D0** | **6 weeks** | **10 weeks** |
| --- | --- | --- | --- | --- |
| **CTRL** | Body Weight | 20.63±0.057 | 22.41±0.487 | 24.71±1.657 |
|  | Liver Weight | - | 0.92±0.002 | 1.307±0.097 |
| **IR** | Body Weight | 20.82±1.772 | 24.9±0.17 | 29.4±0.9 |
|  | Liver Weight | - | 1.41±0.171 | 1.34±0.111 |
