# Supplementary material for: Potential Role of Hedgehog Pathway in Liver Response to Radiation
Source: PLoS One. 2013 Sep 16;8(9):e74141. doi: 10.1371/journal.pone.0074141 (PMC3774612; doi:10.1371/journal.pone.0074141)
Supplement: Table S2 — Liver and body weight in GDC-0449 treatment. (DOCX) [file pone.0074141.s004.docx]

**Supporting Table S2. Liver and body weight in GDC-0449 treatment**

|  | | **D0** | **6 weeks** |
| --- | --- | --- | --- |
| **DMSO** | Body Weight | 20.52±0.389 | 28.04±2.336 |
|  | Liver Weight | - | 1.30±0.160 |
| **DMSO+GDC** | Body Weight | 20.69±0.405 | 27.11±1.613 |
|  | Liver Weight | - | 1.25±0.106 |
| **IR+DMSO** | Body Weight | 20.68±0.465 | 25.26±0.527 |
|  | Liver Weight | - | 1.37±0.114 |
| **IR+GDC** | Body Weight | 20.95±0.249 | 25.02±0.887 |
|  | Liver Weight | - | 1.23±0.063 |
